# Respiratory effects of prone position in COVID-19 acute respiratory distress syndrome differ according to the recruitment-to-inflation ratio: a prospective observational study

**DOI:** 10.1186/s13613-024-01375-2

**Published:** 2024-09-18

**Authors:** Christopher Lai, Rui Shi, Ludwig Jelinski, Florian Lardet, Marta Fasan, Soufia Ayed, Hugo Belotti, Nicolas Biard, Laurent Guérin, Nicolas Fage, Quentin Fossé, Thibaut Gobé, Arthur Pavot, Guillaume Roger, Alex Yhuel, Jean-Louis Teboul, Tai Pham, Xavier Monnet, for the EVALPRO Study group

**Affiliations:** 1 AP-HP, Service de médecine intensive-réanimation, Hôpital de Bicêtre, Hôpitaux Universitaires Paris-Saclay, DMU CORREVE, FHU SEPSIS, Groupe de recherche clinique CARMAS, Université Paris-Saclay, 78 rue du Général Leclerc, 94270 Le Kremlin-Bicêtre, France; 2 Inserm UMR S_999, Pulmonary Hypertension: Pathophysiology and Novel Therapies, University Paris-Saclay, Hôpital Marie Lannelongue, Le Plessis-Robinson, France; 3 Department of Surgery, Dentistry, Gynaecology and Paediatrics, University of Verona, Verona, Veneto Italy; 4 Inserm U1018, Equipe d’Epidémiologie Respiratoire Intégrative, CESP,, Université Paris-Saclay (UVSQ)-Université Paris-Sud, Villejuif, 94807 France

**Keywords:** Airway opening pressure, Heart-lung interactions, Driving pressure, Lung recruitment

## Abstract

**Background:**

Improvements in oxygenation and lung mechanics with prone position (PP) in patients with acute respiratory distress syndrome (ARDS) are inconstant. The objectives of the study were (i) to identify baseline variables, including the recruitment-to-inflation ratio (R/I), associated with a positive response to PP in terms of oxygenation (improvement of the ratio of arterial oxygen partial pressure over the inspired oxygen fraction (PaO_2_/FiO_2_) ≥ 20 mmHg) and lung mechanics; (ii) to evaluate whether the response to the previous PP session is associated with the response to the next session.

**Methods:**

In this prospective, observational, single-center study in patients who underwent PP for ARDS due to COVID-19, respiratory variables were assessed just before PP and at the end of the session. Respiratory variables included mechanical ventilation settings and respiratory mechanics variables, including R/I, an estimate of the potential for lung recruitment compared to lung overinflation.

**Results:**

In 50 patients, 201 PP sessions lasting 19 ± 3 h were evaluated. Neuromuscular blockades were used in 116 (58%) sessions. The PaO_2_/FiO_2_ ratio increased from 109 ± 31 mmHg to 165 ± 65 mmHg, with an increase ≥ 20 mmHg in 142 (71%) sessions. In a mixed effect logistic regression, only pre-PP PaO_2_/FiO_2_ (OR 1.12 (95% CI [1.01–1.24])/every decrease of 10 mmHg, *p* = 0.034) in a first model and improvement in oxygenation at the previous PP session (OR 3.69 (95% CI [1.27–10.72]), *p* = 0.017) in a second model were associated with an improvement in oxygenation with PP. The R/I ratio (*n* = 156 sessions) was 0.53 (0.30–0.76), separating lower- and higher-recruiters. Whereas PaO_2_/FiO_2_ improved to the same level in both subgroups, driving pressure and respiratory system compliance improved only in higher-recruiters (from 14 ± 4 to 12 ± 4 cmH_2_O, *p* = 0.027, and from 34 ± 11 to 38 ± 13 mL/cmH_2_O, respectively, *p* = 0.014).

**Conclusions:**

A lower PaO_2_/FiO_2_ at baseline and a positive O_2_-response at the previous PP session are associated with a PP-induced improvement in oxygenation. In higher-recruiters, lung mechanics improved along with oxygenation. Benefits of PP could thus be greater in these patients.

**Supplementary Information:**

The online version contains supplementary material available at 10.1186/s13613-024-01375-2.

## Introduction

In patients with moderate-to-severe acute respiratory distress syndrome (ARDS), prone positioning (PP) is associated with reduced mortality [1, 2] and is thus recommended when the ratio of the arterial partial pressure of oxygen to the inspired fraction of oxygen (PaO_2_/FiO_2_) is ≤ 150 mmHg [3, 4]. Improvement in survival of such patients is secondary to respiratory effects including homogenization in lung stress and strain, reduced lung overinflation, increased lung recruitment and thus improved ventilation/perfusion matching [5–7]. In addition to these respiratory mechanisms, PP also has some beneficial hemodynamic effects that may play a significant role [5, 8].

Although PP is generally associated with improved oxygenation, this effect is difficult to predict due to the complexity of the determinants of oxygenation and of the effects of PP on both lung and circulation [9–12]. Several studies have investigated whether factors could be identified to predict a positive response to PP. The predictive ability of the response in oxygenation of the previous PP session has not been investigated.

The beneficial effects of PP are likely more related to lung protective effects than effects on oxygenation. However, these effects are again difficult to predict. This may be possible with the recruitment-to-inflation (R/I) ratio. This index does not estimate lung recruitment, but has been proposed for an easy assessment at the bedside of the potential for lung recruitment in patients with ARDS [13]. It might help in setting the level of positive end-expiratory pressure (PEEP) [14–16] or in deciding to apply lung recruitment maneuvers [17]. Whether it could predict the response of lung mechanics induced by PP has been investigated in a small study in COVID-19 patients with ARDS [18], which may require some confirmation.

The objectives of this prospective observational study in ARDS patients were (i) to identify baseline variables, including the R/I ratio, that are associated with a positive response to PP in terms of oxygenation and lung mechanics and (ii) to evaluate whether the response to the previous PP session is associated with the response to the next session.

## Methods

### Study population

This prospective observational cohort study was performed in a 25-bed medical intensive care unit. It was approved by the ethics committee of the French Intensive Care Society (CE SRLF 21–01) and registered at ClinicalTrials.gov (NCT04635267). All patients or close relatives were informed that their data were included in the cohort. It was conducted according to the STROBE guidelines (Additional file 1: Appendix 1).

Patients were eligible if they met the criteria for ARDS [19], were aged ≥ 18 years, were under invasive mechanical ventilation, were monitored with a transpulmonary thermodilution device, according to current guidelines [20], and if attending physicians decided to perform PP, according to current guidelines [4]. The exclusion criteria were the presence of extracorporeal membrane oxygenation (ECMO) and pregnancy. The non-inclusion criteria were the unavailability of the investigators and the necessity of performing PP in an emergency. Several PP sessions per patient could be included.

### Study design and data collection

PEEP was set according to the “Express” protocol [21]. The plateau pressure was measured during a 3-sec end-inspiratory hold of the ventilator. For all measurements, the absence of respiratory effort or asynchrony was carefully checked. Pressure, volume, and flow curves were not continuously recorded. Blood gas samples were collected before the assessment of respiratory parameters. Measurements were performed during volume control with Carescape R860 (General Electrics, Fairfield, CO) or Infinity C500 (Dräger Medical, Lübeck, Germany) ventilators in either the supine semi-recumbent position or in the prone position (bed in the proclive position at 10–12°).

The compliance of the respiratory system (Crs) was calculated as the tidal volume divided by the driving pressure (DP, plateau pressure – total PEEP) [22]. The airway opening pressure (AOP) and R/I were obtained as previously described [13, 23]. AOP was measured using cursors on the ventilator screen by inspecting the pressure–time curve during a low-flow insufflation (6 L/min) starting from a PEEP level of 0. The PEEP level was set at 15 cmH_2_O for at least 15 min. Then, the respiratory rate was decreased to 10 breaths/min to avoid possible intrinsic PEEP, and the expired tidal volume displayed by the ventilator was noted. PEEP was abruptly decreased by 10 cmH_2_O and the expired volume displayed by the ventilator immediately after the maneuver was collected. Finally, plateau pressure at low PEEP was assessed. The recruited lung volume divided by the effective pressure change (depending on the presence of AOP) allows the calculation of the compliance of the recruited lung. The R/I ratio is the ratio between the compliance of the recruited lung and that of the respiratory system at low PEEP [13]. R/I ratios were computed using an online calculator (www.rtmaven.com).

PP sessions were performed as previously described and recommended [3]. During PP, the arms were parallel to the trunk, the abdomen was unsupported, and the face turned to the right or the left side. The bed was placed in the proclive position at 10–12°. Ventilatory settings and respiratory and hemodynamic variables were collected prospectively in the hour before PP, at the end of the PP session just before the patient was returned to the supine position and, when feasible, 6–8 h after the end of the PP session. A detailed study design is provided in the Supplemental material.

### Statistical analysis

Variables are reported as mean ± SD or median (interquartile range), and n (%). Proportions were compared using chi-square and Fisher exact tests, and continuous variables were compared using Student’s t tests, Wilcoxon rank sum tests or paired tests, as appropriate. The correlation coefficients were compared with a z-test on Fisher z-transformed correlation coefficients. Changes in variables over time were assessed by a repeated measures ANOVA model. For pairwise comparisons between different time points (before PP, end of PP and post PP), a Bonferroni correction was applied. Most patients had several PP sessions, leading to a clustered structure of the data. To consider this repeated data collection, we used a mixed effect logistic regression to determine factors associated with the outcomes of interest: level 1 comprised session-related variables, and level 2 comprised patient-related covariates [24, 25].

To assess our two objectives, to identify baseline variables associated with a positive response to PP in terms of oxygenation and lung mechanics and to evaluate whether the response to the previous PP session is associated with the response to the next session, and to identify baseline variables associated with a positive O_2_-response to PP, the O_2_-response was defined as an increase in PaO_2_/FiO_2_ ≥ 20 mmHg during PP (using the arterial blood gas drawn at the end of the PP session, just before turning the patient back to the supine position) [10, 12, 26]. We selected variables a priori based on their clinical relevance or their expected association with the outcomes of interest. In a first model, the following factors were entered: a decrease in PaO_2_/FiO_2_ by 10 mmHg, driving pressure and SAPS II. In a second model, we added the R/I ratio. In a third model, we introduced the O_2_-response from the previous PP session when available. If variables were associated with an O_2_-response with a p value < 0.10 in the univariate regression analysis, they were included in the model. The results are shown as odds ratio (OR) with 95% confidence interval (95% CI).

To analyze the response to PP in terms of oxygenation and the respiratory mechanics depending on the R/I ratio before the PP session, this continuous variable was transformed into a binary variable (higher or lower) on either side of the median value measured in the supine position. A regression model to explain improvement in oxygenation and respiratory mechanics was then performed, including R/I before the considered session and body mass index (BMI) as explanatory covariates. Pearson’s correlation was used to test the relationship between the R/I ratio and changes in respiratory variables in the supine position and in PP.

Assuming an incidence of 25% of O_2_-non-response with PP [10, 11], and considering that 10 events per variable would be necessary to perform the logistic regression analysis with 5 factors [27], we calculated that 200 PP sessions should be analyzed in the study. Considering that each patient would undergo 4 PP sessions [1], 50 patients were included.

A p-value < 0.05 was considered significant. The statistical analysis was performed using MedCalc 19.2.1 software (MedCalc Software Ltd., Ostend, Belgium) or R 4.21 (R Foundation for Statistical Computing, Vienna, Austria, http://www.R-project.org*).*

## Results

### Study population

Between January and May 2021, among the 60 eligible patients, 50 were included. In these patients, 201 PP sessions were recorded prospectively (Supplementary Figure S1), with a median of 3 (2–6) sessions per patient and a maximum of 11 sessions in one patient. The first PP was recorded in 44/50 (88%) patients (Table 1). Neuromuscular blocking agents (NMBA) were used during 116 (58%) sessions. Inhaled nitric oxide was used in 3 (6%) patients and during 4 (2%) of the analyzed sessions (Supplementary Table S1). Severe acute cor pulmonale was observed in 2 (4%) patients. Veno-venous ECMO was implanted secondarily in 5 (10%) patients for refractory hypoxemia and follow-up was stopped in these patients. The mortality rates on day-30 and day-90 were 40% and 52%, respectively.

**Table 1:** Patient characteristics

Gender, male/female	40/10
Age, years	63 ± 9
Height, cm	172 ± 9
Weight, kg	88 ± 14
Body mass index, kg/m^2^	29.7 ± 5.5
Medical history Hypertension Diabetes mellitus Immunosuppression	21 (42) 12 (24) 4 (8)
SAPS II	35 ± 12
Time from hospital to ICU admission, days	1 (0–3)
Time from ICU admission to intubation, days	1 (0–2)
Time from intubation to first prone position session, days (*n* = 44)	1 (0–3)
Pulmonary embolism at admission, N	7 (14)
Adjunctive therapies, N Dexamethasone Tocilizumab Bevacizumab	50 (100) 14 (28) 2 (4)
ICU length of stay, days	26 ± 18
ICU mortality, N	24 (48)

*N* = 50. The results are expressed as numbers, numbers (%) or mean ± SD

*ICU* intensive care unit, *SAPS* simplified acute physiology score

When considering all sessions, the severity of ARDS at the time of PP was moderate and severe in 114 (57%) and 87 (43%) sessions, respectively. The pre-PP PaO_2_/FiO_2_ was ≤ 150 mmHg in 181 (91%) sessions. One-hundred-sixty-one (80%) sessions were performed during the first two weeks following hospital admission, 33 (16%) during the third week and seven (3%) during the fourth week after hospital admission. The tidal volume was 6.1 ± 0.3 mL/kg of predicted body weight, and the respiratory rate was 29 ± 4/min. The other respiratory variables prior to PP are reported in Table 2.

**Table 2:** Respiratory variables at different study times

	Pre-PP		PP		Post-PP		ANOVA
	*n*		*n*		*n*		*p*
PEEPt, cmH_2_O All O_2_-responders O_2_-nonresponders Lower-recruiters Higher-recruiters	201 142 59 75 81	14 ± 3 14 ± 3 14 ± 3 14 ± 3 15 ± 3	201 142 59 75 81	14 ± 3 14 ± 3 14 ± 3 14 ± 3 15 ± 3	137 99 38 49 52	14 ± 3 14 ± 3 14 ± 3 14 ± 3 15 ± 3	0.652 0.288 0.444 0.889 0.643
Pplat, cmH_2_O All O_2_-responders O_2_-nonresponders Lower-recruiters Higher-recruiters	201 142 59 75 81	29 ± 4 29 ± 4 29 ± 4 29 ± 4 28 ± 3	201 142 59 75 81	28 ± 4 28 ± 4 28 ± 4^$^ 28 ± 3 27 ± 4	137 99 38 49 52	29 ± 4 28 ± 3 30 ± 4 29 ± 4 28 ± 3	0.560 0.567 0.003 0.949 0.965
Driving pressure, cmH_2_O All O_2_-responders O_2_-nonresponders Lower-recruiters Higher-recruiters	201 142 59 75 81	15 ± 5 15 ± 5 16 ± 6 15 ± 5 14 ± 4	201 142 59 75 81	14 ± 5* 14 ± 5 15 ± 6 15 ± 5 12 ± 4*	137 99 38 49 52	15 ± 6 14 ± 6 16 ± 7 15 ± 5 13 ± 4	0.018 0.129 0.053 0.596 0.008
Crs, mL/cmH_2_O All O_2_-responders O_2_-nonresponders Lower-recruiters Higher-recruiters	201 142 59 75 81	30 ± 11 30 ± 10 31 ± 13 30 ± 10 34 ± 11	201 142 59 75 81	33 ± 13*^$^ 33 ± 12 32 ± 15^$^ 30 ± 10 38 ± 13*^$^	137 99 38 49 52	31 ± 9 32 ± 9 29 ± 8 30 ± 8 33 ± 9	0.001 0.569 0.021 0.927 0.002
PaO_2_/FiO_2_, mmHg All O_2_-responders O_2_-nonresponders Lower-recruiters Higher-recruiters	201 142 59 75 81	109 ± 31 106 ± 31 115 ± 30 115 ± 33 107 ± 29	201 142 59 75 81	163 ± 65*^$^ 185 ± 61*^$^ 110 ± 32 161 ± 61*^$^ 168 ± 72*^$^	137 99 38 51 51	135 ± 54^¤^ 143 ± 53^¤^ 113 ± 51 129 ± 49 139 ± 62^¤^	< 0.001 < 0.001 0.486 < 0.001 < 0.001
AOP, cmH_2_O All O_2_-responders O_2_-nonresponders Lower-recruiters Higher-recruiters	156 106 50 75 81	0 (0–6) 1 (0–6) 0 (0–6) 0 (0–6) 1 (0–6)	158 113 45 59 69	2 (0–6) 2 (0–5) 2 (0–5) 2 (0–5) 2 (0–7)	107 78 29 40 45	3 (0–6) 3 (0–6) 4 (2–6) 3 (0–6) 3 (0–6)	0.375 0.799 0.232 0.406 0.803
R/I All O_2_-responders O_2_-nonresponders Lower-recruiters Higher-recruiters	156 106 50 75 81	0.53 (0.31–0.81) 0.53 (0.29–0.76) 0.53 (0.40–0.85) 0.30 (0.00–0.43) 0.76 (0.61–1.16)	158 113 45 59 69	0.51 (0.30–0.76) 0.48 (0.30–0.70) 0.61 (0.36–0.83) 0.45 (0.27–0.71)* 0.57 (0.40–0.86)*	107 78 29 40 45	0.54 (0.27–0.85) 0.54 (0.27–0.88) 0.53 (0.26–0.79) 0.45 (0.04–0.88)^¤^ 0.62 (0.46–0.84)	0.344 0.241 0.247 0.006 0.019

ANOVA analysis of variance; AOP airway opening pressure; Crs respiratory system compliance; FiO_2_ inspired fraction in oxygen; PEEPt total positive end-expiratory pressure; PaO_2_ arterial partial pressure in oxygen; PP prone position; Pplat plateau pressure; R/I recruitment-to-inflation ratio

“Pre-PP”: ≤1 h before the PP; “PP”: at the end of the PP session; “Post-PP”: 6 to 8 h after returning to the supine position

* *p* < 0.05 pre-PP vs. PP, ^$^*p* < 0.05 PP vs. Post-PP, ^¤^*p* < 0.05 pre-PP vs. post-PP

### Baseline variables associated with an improvement in oxygenation during PP

Considering all sessions, 142 (71%) were O_2_-responsive, i.e., were accompanied by an increase in PaO_2_/FiO_2_ ≥ 20 mmHg at the end of the PP session compared to before the PP session. Among the 31 patients with ≥ 3 PP sessions, the five who were O_2−_responders to all sessions survived on day-90, and the two who were O_2_-non-responders to all sessions died before day-90 (Supplementary Figure S2).

Univariate analysis showed that PaO_2_ before PP was higher in O_2_-responders than in O_2_-non-responders (Supplementary Table S1). The PaO_2_/FiO_2_ ratio before PP was not different between groups (106 ± 31 mmHg vs. 115 ± 30 mmHg, respectively, *p* = 0.081). No other pre-PP respiratory or hemodynamic variables differed between O_2_-responders and O_2_-non-responders (Supplementary Table S1). When considering only the first PP session (*n* = 44), no variable was associated with an improvement in oxygenation during PP. DP, Crs, the PaO_2_/FiO_2_ ratio and the R/I ratio were similar pre-PP. Results were similar in patients with and without NMBA (data not shown).

The mixed effect logistic regression showed that a lower baseline PaO_2_/FiO_2_ was associated with an O_2_-response during the PP session (Table 3). The R/I before PP was not associated with an O_2_-response (OR 1.15; 95% CI [0.67–1.54], *p* = 0.942) and neither was the timing in days since intubation (OR 0.97; 95% CI [0.89–1.05], *p* = 0.446) (Supplementary Tables S2 and S3).

**Table 3:** Mixed effect logistic regression analysis for factors associated with an improvement in oxygenation in the prone position

	Odds ratio	95% Confidence interval	*p*
PaO_2_/FiO_2_ (/each decrease by 10 mmHg)	1.120	1.009–1.244	0.034
Driving pressure (cmH_2_O)	1.029	0.966–1.096	0.375
SAPS II	1.008	0.982–1.036	0.533

*n* = 201 sessions

FiO_2_: inspired fraction in oxygen; PaO_2_: arterial partial pressure in oxygen; SAPS: simplified acute physiology score

### Baseline variables associated with an improvement in lung mechanics during PP

**Fig. 1:**
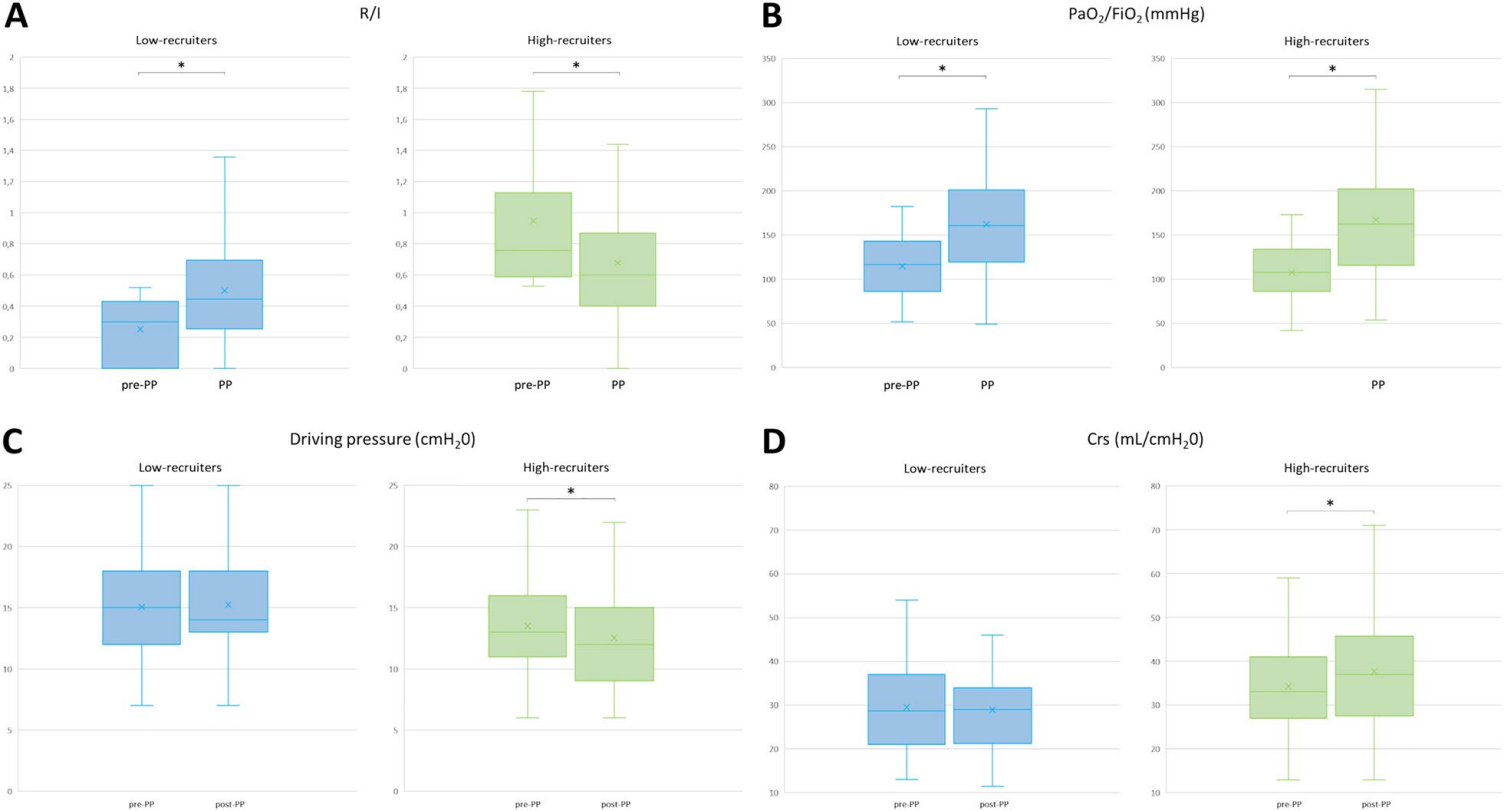
Changes in lung mechanics and oxygenation in the prone position according to the higher- and lower-recruiter profiles. **A** change in R/I; **B** change in PaO_2_/FiO_2_; **C** change in driving pressure; **D** change in respiratory system compliance *p < 0.05: PP vs. Pre-PP FiO_2_: fraction inspired in oxygen; PaO_2_: arterial partial pressure in oxygen; R/I: recruitment-to-inflation ratio

The AOP could be measured before 156 (78%) PP sessions performed in 49 patients (Table 2). An AOP was absent (i.e., 0 cmH_2_O) before 81 (52%) sessions. In the 75 (48%) sessions before which it was present (i.e., ≥ 1 cmH_2_O), the value of AOP was 6 (5–9) cmH_2_O. Among the 156 sessions before which AOP was measured, the median R/I ratio was 0.53 (0.31–0.79), separating higher- and lower-recruiters (R/I ≥ 0.53 and < 0.53, respectively). The PP-induced increase in the PaO_2_/FiO_2_ ratio was similar in higher- and lower-recruiters (*p* = 0.191) (Fig. 1).

In higher-recruiters, the DP decreased during the PP session, while it remained unchanged in lower-recruiters (Table 2; Fig. 1). The mixed effect logistic regression showed that a higher-recruiter status at baseline (OR 4.96; 95% CI [1.84–13.37], *p* = 0.002), a higher pre-PP DP (OR 1.43; 95% CI [1.20–1.71]/cmH_2_O, *p* < 0.001) and a higher BMI (OR 1.18; 95% CI [1.05–1.32]/kg.m^−2^, *p* = 0.004) were associated with a decrease in DP > 0 cmH_2_O during the PP session (Supplementary Table S4). The correlation between R/I before PP and the change in DP during PP was significant (*r*=− 0.31 (− 0.46; − 0.14), *p* < 0.001) and was not different between higher- and lower-recruiters.

In higher-recruiters, Crs increased during the PP session, while it remained unchanged in lower-recruiters (Table 2; Fig. 1). The mixed effect logistic regression showed that a higher-recruiter status (OR 6.95; 95% CI [2.30–20.99], *p* < 0.001), a lower Crs before the PP session (OR 0.85; 95% CI [0.78–0.93] /cmH_2_O, *p* < 0.001) and a higher BMI (OR 1.17; 95% CI [1.03–1.32]/kg.m^−2^, *p* = 0.016) were associated with an increase in Crs during the PP session (Supplementary table S5). The correlation between R/I before PP and the change in Crs during PP was significant (*r* = 0.37 (0.21; 0.51), *p* < 0.001) and was not different between higher- and lower-recruiters. In higher-recruiters (*n* = 69), the R/I decreased between before and the end of PP while it increased in lower-recruiters (Table 2; Fig. 1).

Association of the response in oxygenation of a PP session with the response to the next session.

For 137 sessions performed in 38 patients in whom ≥ 2 PP sessions had been performed, the O_2_-response of the previous session could be analyzed. For the 101 sessions in which oxygenation improved, there was a positive O_2_-response at the previous PP session in 75 (74%) cases. Conversely, in the other 36 sessions in which oxygenation did not improve, there was a positive O_2_-response at the previous PP session in 16 (44%) sessions (Supplementary Figure S2). The mixed effect logistic regression showed that a positive O_2_-response at the previous PP session was significantly associated with a significant improvement in oxygenation during the current session (Table 4).

**Table 4:** Mixed effect logistic regression analysis for factors associated with an improvement in oxygenation in the prone position, including the oxygen response at the previous session

	Odds ratio	95% Confidence interval	*p*
PaO_2_/FiO_2_ (/each decrease by 10 mmHg)	1.127	0.970–1.310	0.117
Driving pressure (cmH_2_O)	1.065	0.956–1.043	0.248
SAPS II	1.005	0.967–1.043	0.814
R/I ratio	0.986	0.618–1.574	0.954
O_2_-response at previous PP session	3.690	1.270-10.718	0.017

*N* = 106 sessions

FiO_2_ inspired fraction in oxygen; PaO_2_ arterial partial pressure in oxygen; PP prone position; R/I recruitment-to-inflation ratio; SAPS simplified acute physiology score

### Return to supine position

The changes induced by returning the patient to the supine position were obtained for 137 sessions performed in 35 patients. They were assessed 7 (6–8) hours after the PP session (Supplementary Table S6). The R/I after returning the patient to the supine position was obtained in 102 sessions and was 0.53 (0.32–0.69), comprising 51 (50%) sessions in lower-recruiters and 51 (50%) in higher-recruiters. The R/I values at the three timepoints, i.e., before PP, at the end of PP and after PP, were obtained in 76 sessions (Supplementary Table S7).

After PP, the PaO_2_/FiO_2_ ratio decreased in both lower- and higher-recruiters. Compared to the end of the PP session, DP and Crs decreased in higher-recruiters, but they did not change in lower-recruiters, while the R/I did not change in lower- or in higher-recruiters (Supplementary Table S6 and Figure S3).

## Discussion

In this prospective observational study in patients with COVID-19-related ARDS, we found that (i) a lower PaO_2_/FiO_2_ ratio before PP was associated with a positive O_2_-response during PP; (ii) a positive O_2_-response during the previous PP session was associated with the O_2_-response during the following session; and (iii) a higher potential of lung recruitability at baseline was associated with an improvement in lung mechanics during PP.

### Oxygenation improvement

Although PP was widely used to treat severely ill patients during the COVID-19 pandemic [28, 29], a reduction in its use was observed later [30]. One reason for this underuse of PP in daily practice might be that its positive benefit/risk ratio is not fully perceived by staff physicians and nurses, who are often overwhelmed by the burden of daily workload. Although safe, PP is time-consuming and staff must be trained and numerous. Also, pressure sores can develop secondary to long-lasting sessions ≥ 16 h [31]. Therefore, predicting the response to PP might be helpful in selecting patients for whom it should be beneficial.

First, we found that the lower the baseline PaO_2_/FiO_2_ ratio, the higher the likelihood of a positive O_2_-response. The lower the baseline PaO_2_/FiO_2_, the greater the likelihood of a positive O_2_-response. A similar result has been reported in other studies in COVID-19-related ARDS [32, 33]. This finding is also consistent with the fact that in non-COVID-19 ARDS, PP is beneficial not in all ARDS forms but only in moderate-to-severe cases [2]. No other respiratory or hemodynamic variables recorded before the PP session were associated with an improvement in oxygenation during the session, particularly lung mechanics variables and the R/I ratio.

Second, we found that an improvement in oxygenation at the previous PP session was associated with a positive O_2_-response at the current session. Thus, in the absence of contra-indications, PP should be considered with little hesitation in patients in whom the previous session has significantly improved oxygenation. Nevertheless, a negative O_2_-response at the previous PP session should not necessarily discount PP, as 44% of PP sessions induced an improvement in oxygenation, whereas there was no O_2_-response at the previous session. However, deciding to perform new PP sessions based on the oxygenation response during the previous PP is debatable. Previous studies showed that this response in oxygenation does not influence the outcome, contrary to the response in lung mechanics, even though different results were observed in the specific population of COVID-19 patients with ARDS [34].

These results may be interesting, as most studies evaluating the effects of PP analyzed only one session, usually the first one [10, 14, 18, 32, 35]. In a retrospective study in patients with COVID-19-related ARDS experiencing ≥ 2 PP sessions, Weiss et al. found that an improvement in oxygenation during the second PP session was associated with a better outcome compared to those with no positive O_2_-response [34]. However, these authors only assessed the first three PP sessions and did not evaluate the impact of the O_2_-response of the previous session on the next one.

### Lung recruitability and prone position

The R/I ratio has been proposed as an easy tool to assess lung recruitability at the bedside [13]. It significantly correlates with the proportion of lung tissue recruited by the change in PEEP, assessed through computed tomography scan [35] or electrical impedance tomography [14]. The R/I ratio also correlates with the improvement in Crs secondary to lung recruitment maneuvers [17]. Accordingly, we found that the improvement in DP and Crs induced by PP occurred only in higher-recruiters defined by a higher R/I, whereas in lower-recruiters, PP improved oxygenation without changing lung mechanics. We defined higher- and lower-recruiters by considering the median of measurements rather than a given threshold from previous literature. Although this may be criticized, it avoids the limitation of variability in the measurement of the R/I ratio between ventilators of different brands [36].

Our results are consistent with those of Cour et al., who reported that, during PP, R/I decreases in higher-recruiters and increases in lower-recruiters in patients with COVID-19 and ARDS [18]. Interestingly, the R/I increased less in that study than in ours, perhaps because it was assessed after only 2 h of PP, while we evaluated the R/I change at the end of the PP session. This timing issue may also explain why Taenaka et al. reported no change in R/I with PP when measured only 30 min after starting PP [14].

This result might have important implications for how R/I can be used to personalize ventilator settings in patients with ARDS undergoing PP. In higher-recruiters, if lung mechanics improve and R/I decreases with PP, this may reflect effective lung recruitment. The level of PEEP could be lowered to avoid overinflation, decrease lung strain and decrease the risk of ventilator-induced lung injury. In lower-recruiters, if R/I increases with PP, it may reflect a gain in lung recruitability. This might be an argument to test a PEEP increase, as the patient might then be assessed as a higher-recruiter, or to extend the PP session duration. Indeed, long sessions appear feasible and safe [37–39] and might be effective in some patients [37, 40].

The association we observed between R/I before PP and the improvement in lung mechanics during PP may be clinically relevant. On the one hand, it has been shown that the improvement in outcome with PP in patients with ARDS was not associated with the improvement in oxygenation [12]. On the other hand, even though changes in Crs and DP may also be difficult to analyze, as PP can have different (and opposite) effects on lung and chest wall compliances, improvements in these respiratory parameters may still be beneficial. Indeed, Guérin et al. found in a secondary analysis of the PROSEVA [1] and ACURASYS [41] trials that improvements in DP and Crs were associated with survival in patients with ARDS [42]. As this latter study demonstrated an improvement in day-90 survival per each unit of DP on day-1, we chose to define a PP-induced decrease in DP ≥ 1 cmH_2_O as significant, and consequently did the same for Crs increases ≥ 1 mL/cmH_2_O. This probably emphasizes that the beneficial effects of PP on prognosis are likely due to minimizing ventilator-induced lung injury rather than only improving oxygenation and that non-response in terms of oxygenation should not be a disincentive in proposing further PP sessions to the patient.

Our study has several limitations. First, it was a single-center study, which may limit the generalizability of the results. However, only two patients with no ECMO screened during the study period were not included in the analysis and PP was performed quasi-systematically when PaO_2_/FiO_2_ was < 150 mmHg in included patients. Second, we did not assess factors associated with mortality because of this limited sample size, but this was not the purpose of the study. Third, we included only patients with COVID-19-related ARDS due to the inclusion period. Such forms of ARDS may be a specific entity, with higher Crs than other forms [43, 44], though this is debated [45]. Moreover, pulmonary fibrosis that may occur in such patients could impact the PP response. Nevertheless, the mixed effect logistic regression model we used did not evidence an influence of time on the effects of PP on lung mechanics and oxygenation. Fourth, the definition of the O_2_-response as an increase in PaO_2_/FiO_2_ ≥ 20 mmHg is arbitrary. However, this threshold proposed by Chatte et al. in 1997 [11] has been used in several subsequent studies [10, 12, 26]. Fifth, all patients were not paralyzed during PP sessions. Not only may NMBA allow more volume expansion by preventing expiratory muscle activity, but there may also be a synergistic effect of NMBA with PP. However, lung mechanics were similar during PP sessions with and without NMBA. Finally, no specific adaptation of PEEP in the supine or prone position was performed, though this may maximize recruitment. However, this allowed us to evaluate the specific involvement of the R/I ratio.

In conclusion, we found that the lower the PaO_2_/FiO_2_ ratio before a PP session, the greater the likelihood of improving oxygenation with PP. The O_2_-response during a PP session was also more likely if the previous PP session induced a positive O_2_-response. Whereas oxygenation improved during PP in both higher- and lower-recruiters, as defined according to the R/I, lung mechanics improved only in higher-recruiters.

## Supplementary Information

Supplementary Material 1.

## Data Availability

The datasets used and/or analyzed during the current study are available from the corresponding author upon reasonable request.

## Abbreviations

AOP: Airway opening pressure
ARDS: Acute respiratory distress syndrome
Crs: Compliance of the respiratory system
DP: Driving pressure
ECMO: Extracorporeal membrane oxygenation
FiO_2_: The inspired fraction of oxygen
NMBA: Neuromuscular blocking agents
PaO_2_: Arterial oxygen partial pressure
PEEP: Positive end-expiratory pressure
PP: Prone positioning
R/I: Recruitment-to-inflation ratio
