# Supplementary material for: Respiratory effects of prone position in COVID-19 acute respiratory distress syndrome differ according to the recruitment-to-inflation ratio: a prospective observational study
Source: Ann Intensive Care. 2024 Sep 18;14:146. doi: 10.1186/s13613-024-01375-2 (PMC11411043; doi:10.1186/s13613-024-01375-2)
Supplement: Supplementary file 1 — Supplementary Material 1. [file 13613_2024_1375_MOESM1_ESM.docx]

**SUPPLEMENTAL MATERIAL**

**Respiratory effects of prone position in acute respiratory distress syndrome differ according to the recruitment-to-inflation ratio: a prospective observational study.**

Christopher LAI, Rui SHI, Ludwig JELINSKI, Florian LARDET, Marta FASAN, Soufia AYED, Hugo BELOTTI, Nicolas BIARD, Laurent GUÉRIN, Nicolas FAGE, Quentin FOSSÉ, Thibault GOBÉ, Arthur PAVOT, Guillaume ROGER, Alex YUEL, Jean-Louis TEBOUL, Tai PHAM, Xavier MONNET for the EVALPRO Study group.

Table of contents

[Supplemental methods 3](#_Toc170860776)

[*Procedure for assessment of respiratory mechanics* 3](#_Toc170860777)

[Appendix 1. STROBE Statement 4](#_Toc170860778)

[Table S1. Respiratory and hemodynamic variables at baseline according to the oxygenation response during the prone position session 6](#_Toc170860779)

[Table S2. Mixed effect logistic regression analysis for factors, including the recruitment-to-inflation ratio, associated with an improvement in oxygenation with prone position as a dependent factor and the recruitment-to-inflation ratio as an independent factor. 7](#_Toc170860780)

[Table S3. Mixed effect logistic regression analysis for factors associated with an improvement in driving pressure. 7](#_Toc170860781)

[Table 4. Mixed effect logistic regression analysis for factors associated with an improvement in respiratory system compliance. 8](#_Toc170860782)

[Table S5. Respiratory and hemodynamic variables at baseline, end of prone position and post prone position. 9](#_Toc170860783)

[Table S6. Changes in respiratory variables with prone position and after prone position 10](#_Toc170860784)

[Table S7. Mixed effect logistic regression analysis for factors, associated with an improvement in oxygenation with prone position, including duration of mechanical ventilation before the session. 10](#_Toc170860785)

[Figure S1. Flowchart 11](#_Toc170860786)

[Figure S2. Alluvial diagram of patients’ movements between O2-response with prone position 12](#_Toc170860787)

[Figure S3. Changes in airway opening pressure between two consecutives prone position sessions 13](#_Toc170860788)

[Figure S4. Changes in lung mechanics and oxygenation with prone position and post prone position according to higher- and lower-recruiter profile. 14](#_Toc170860789)

# Supplemental methods

## *Procedure for assessment of respiratory mechanics*

1) Expiratory pause for 5 sec for total PEEP assessment when the flow is zero

2) Inspiratory pause for 3 sec. Pplat is was assessed in the first 0.5 sec when the flow was zero.

3) Assessment of AOP when feasible, starting insufflation at PEEP 0 cmH_2_0.

4) Setting PEEP at 15 cmH_2_O (or 5 cmH_2_O above the AOP, whether AOP >10cmH_2_O) for ≥15 minutes

5) Assessment of the R/I ratio when feasible

5-1) Decreasing the flow to 6L/min and respiratory rate to 10/min to avoid auto-PEEP

5-2) Decreasing PEEP by 10 cmH_2_0 and assessing the volume expired with the maneuver

5-3) Assessing plateau pressure at the new low level of PEEP

6) Calculation of the R/I ratio using the online calculator www.rtmaven.com

# Appendix 1. STROBE Statement

|  | Item No | Recommendation | Page No |
| --- | --- | --- | --- |
| **Title and abstract** | 1 | (*a*) Indicate the study’s design with a commonly used term in the title or the abstract | 1 |
|  |  | (*b*) Provide in the abstract an informative and balanced summary of what was done and what was found | 2 |
| Introduction | | | |
| Background/rationale | 2 | Explain the scientific background and rationale for the investigation being reported | 4 |
| Objectives | 3 | State specific objectives, including any prespecified hypotheses | 4 |
| Methods | | | |
| Study design | 4 | Present key elements of study design early in the paper | 5 |
| Setting | 5 | Describe the setting, locations, and relevant dates, including periods of recruitment, exposure, follow-up, and data collection | 5-7 |
| Participants | 6 | (*a*) Give the eligibility criteria, and the sources and methods of selection of participants. Describe methods of follow-up | 5 |
|  |  | (*b*) For matched studies, give matching criteria and number of exposed and unexposed |  |
| Variables | 7 | Clearly define all outcomes, exposures, predictors, potential confounders, and effect modifiers. Give diagnostic criteria, if applicable | 5;6 |
| Data sources/ measurement | 8* | For each variable of interest, give sources of data and details of methods of assessment (measurement). Describe comparability of assessment methods if there is more than one group | 5;6 |
| Bias | 9 | Describe any efforts to address potential sources of bias | 5 |
| Study size | 10 | Explain how the study size was arrived at | 7 |
| Quantitative variables | 11 | Explain how quantitative variables were handled in the analyses. If applicable, describe which groupings were chosen and why | 5;6 |
| Statistical methods | 12 | (*a*) Describe all statistical methods, including those used to control for confounding | 6;7 |
|  |  | (*b*) Describe any methods used to examine subgroups and interactions |  |
|  |  | (*c*) Explain how missing data were addressed |  |
|  |  | (*d*) If applicable, explain how loss to follow-up was addressed |  |
|  |  | (*e*) Describe any sensitivity analyses |  |
| Results | | |  |
| Participants | 13* | (a) Report numbers of individuals at each stage of study—eg numbers potentially eligible, examined for eligibility, confirmed eligible, included in the study, completing follow-up, and analysed | 7 |
|  |  | (b) Give reasons for non-participation at each stage | 7 |
|  |  | (c) Consider use of a flow diagram | Figure S1 |
| Descriptive data | 14* | (a) Give characteristics of study participants (eg demographic, clinical, social) and information on exposures and potential confounders | 7-8 table 1 |
|  |  | (b) Indicate number of participants with missing data for each variable of interest | Table S1 |
|  |  | (c) Summarise follow-up time (eg, average and total amount) | 8 |
| Outcome data | 15* | Report numbers of outcome events or summary measures over time | 7-9 |

| Main results | 16 | (*a*) Give unadjusted estimates and, if applicable, confounder-adjusted estimates and their precision (eg, 95% confidence interval). Make clear which confounders were adjusted for and why they were included | 7-9 |
| --- | --- | --- | --- |
|  |  | (*b*) Report category boundaries when continuous variables were categorized | 8 |
|  |  | (*c*) If relevant, consider translating estimates of relative risk into absolute risk for a meaningful time period | NA |
| Other analyses | 17 | Report other analyses done—eg analyses of subgroups and interactions, and sensitivity analyses | 10 |
| Discussion | | | |
| Key results | 18 | Summarise key results with reference to study objectives | 11 |
| Limitations | 19 | Discuss limitations of the study, taking into account sources of potential bias or imprecision. Discuss both direction and magnitude of any potential bias | 13-14 |
| Interpretation | 20 | Give a cautious overall interpretation of results considering objectives, limitations, multiplicity of analyses, results from similar studies, and other relevant evidence | 11-13 |
| Generalisability | 21 | Discuss the generalisability (external validity) of the study results | 13 |
| Other information | | | |
| Funding | 22 | Give the source of funding and the role of the funders for the present study and, if applicable, for the original study on which the present article is based | 16 |

# Table S1. Respiratory and hemodynamic variables at baseline according to the oxygenation response during the prone position session

|  | All sessions  N=201 | O_2_-responders  N= 142 | O_2_-non-responders  N=59 | p-value |
| --- | --- | --- | --- | --- |
| Respiratory variables | | | | |
| FiO_2_, % | 75±18 | 77±17 | 72±18 | 0.348 |
| Vt, mL/kg | 6.1±0.3 | 6.1±0.3 | 6.1±0.3 | 0.312 |
| RR, /min | 29±4 | 29±4 | 30±4 | 0.154 |
| PEEP, cmH_2_0 | 13±3 | 13±3 | 13±3 | 0.853 |
| PEEPt, cmH_2_0 | 14±3 | 14±3 | 14±3 | 0.831 |
| Pplat, cmH_2_0 | 29 (26-31) | 28 (26-30) | 29 (27-31) | 0.219 |
| Driving pressure, cmH_2_0 | 15±5 | 15±5 | 16±6 | 0.373 |
| Mechanical power, L/min (n=187) | 38 (32-46) | 38 (32-45) | 40 (33-49) | 0.110 |
| Crs, mL/cmH_2_0 | 31±11 | 30±10 | 31±13 | 0.812 |
| pH | 7.38±0.06 | 7.38±0.06 | 7.37±0.07 | 0.615 |
| pCO2, mmHg | 45±7 | 45±8 | 46±7 | 0.232 |
| paO_2_, mmHg | 78±17 | 76±16 | 82±19 | 0.043 |
| paO_2_/FiO_2_, mmHg | 109±31 | 106±31 | 115±30 | 0.081 |
| AOP (N= 156) | 0 (0-6) | 0 (0-6) | 1 (0-6) | 0.562 |
| R/I (N=156) | 0.53 (0.30-0.76) | 0.53 (0.30-0.76) | 0.53 (0.38-0.85) | 0.375 |
| Hemodynamic variables | | | | |
| FC | 79±20 | 80±20 | 77±19 | 0.518 |
| PAS | 131±25 | 132±19 | 129±20 | 0.576 |
| PAD | 65±12 | 65±13 | 65±10 | 0.881 |
| PAM | 88±16 | 88±17 | 88±13 | 0.728 |
| CVP (N= 157) | 11±4 | 11±4 | 12±4 | 0.408 |
| PPV | 7 (5-12) | 7 (5-12) | 7 (5-10) | 0.445 |
| CI | 3.0±0.9 | 3.0±0.9 | 3.1±1.1 | 0.308 |
| GEDVI | 800±199 | 793±196 | 811±208 | 0.573 |
| EVLWi | 19±6 | 19±6 | 19±5 | 0.782 |
| PVPi | 3.7±1.2 | 3.7±1.2 | 3.6±1.1 | 0.393 |
| CFI | 4.2±1.3 | 4.1±1.2 | 4.3±1.4 | 0.398 |
| Norepinephrine (N) | 153 (76) | 104 (73) | 49 (83) | 0.516 |
| Norepinephrine dosage | 0.17 (0.09-0.33) | 0.21 (0.11-0.34) | 0.13 (0.06-0.29) | 0.511 |
| Lactate | 1.7 (1.2-2.3) | 1.6 (1.2-2.1) | 1.8 (1.2-2.4) | 0.313 |
| NMBA | 115 (57) | 81 (58) | 34 (57) | 0.939 |
| NO | 4 (2) | 3 (3) | 1 (2) |  |

Values are expressed as number (%), mean±SD or median (interquartile range).

p values refer to the comparison between O_2_-responders and O_2_-non-responders.

AOP: airway opening pressure; CFI: cardiac function index; CI: cardiac index; Crs: respiratory system compliance; CVP: central venous pressure; DAP: diastolic arterial pressure; EVLWi: extravascular lung water index; FiO_2_: inspired fraction in oxygen; GEDVi: global end-diastolic volume index; HR: heart rate; MAP: mean arterial pressure; NMBA: neuromuscular blockade agents; paCO_2_: partial arterial pressure in carbon dioxide; PEEP: positive end-expiratory pressure: PEEPt: total positive end-expiratory pressure; paO_2_: partial arterial pressure in oxygen; Pplat: plateau pressure; PPV: pulse pressure variation; PVPI: pulmonary vascular resistance index; RR: respiratory rate; R/I; recruitment-to-inflation ratio; SAP: systolic arterial pressure; Vt: tidal volume.

# Table S2. Mixed effect logistic regression analysis for factors, including the recruitment-to-inflation ratio, associated with an improvement in oxygenation with prone position as a dependent factor and the recruitment-to-inflation ratio as an independent factor.

| **N=156 sessions** | **Odds ratio** | **95% confidence interval** | **p** |
| --- | --- | --- | --- |
| **PaO_2_/FIO_2_ (/each decrease by 10mmHg)** | 1.130 | 1.005 -1.270 | 0.040 |
| **Driving pressure (mmHg)** | 1.024 | 0.942-1.113 | 0.580 |
| **SAPS II** | 1.012 | 0.982-1.043 | 0.438 |
| **R/I ratio** | 1.015 | 0.668-1.543 | 0.942 |

FiO_2_: inspired fraction in oxygen; PaO_2_: partial arterial pressure in oxygen; R/I: recruitment-to-inflation; SAPS: simplified acute physiology score.

# Table S3. Mixed effect logistic regression analysis for factors, associated with an improvement in oxygenation with prone position, including duration of mechanical ventilation before the session.

| **N=201 sessions** | **Odds ratio** | **95% confidence interval** | **p** |
| --- | --- | --- | --- |
| **PaO_2_/FIO_2_ (/each decrease by 10mmHg)** | 1.177 | 1.022-1.355 | 0.023 |
| **Driving pressure (mmHg)** | 0.961 | 0.865-1.067 | 0.455 |
| **SAPS II** | 0.984 | 0.939-1.031 | 0.488 |
| **Duration of mechanical ventilation (days)** | 0.967 | 0.888-1.054 | 0.446 |

FiO_2_: inspired fraction in oxygen; PaO_2_: partial arterial pressure in oxygen; SAPS: simplified acute physiology score.

# Table S4. Mixed effect logistic regression analysis for factors associated with an improvement in driving pressure.

| **N=156 sessions** | **Odds ratio** | **95% confidence interval** | **p** |
| --- | --- | --- | --- |
| **PaO_2_/FIO_2_ (/each decrease by 10mmHg)** | 1.038 | 0.900 -1.196 | 0.606 |
| **Driving pressure (mmHg)** | 1.431 | 1.197-1.713 | <0.001 |
| **BMI (kg/m^2^)** | 1.181 | 1.054-1.322 | 0.004 |
| **High-recruiter status** | 4.956 | 1.837-13.366 | 0.002 |

BMI: body mass index; FiO_2_: inspired fraction in oxygen; PaO_2_: partial arterial pressure in oxygen.

# Table S5. Mixed effect logistic regression analysis for factors associated with an improvement in respiratory system compliance.

| **N=156 sessions** | **Odds ratio** | **Confidence interval 95%** | **p** |
| --- | --- | --- | --- |
| **PaO_2_/FIO_2_ (/each decrease by 10mmHg)** | 1.062 | 0.910 -1.240 | 0.443 |
| **Crs (mmHg)** | 0.853 | 0.784-0.929 | <0.001 |
| **BMI (kg/m^2^)** | 1.165 | 1.029-1.318 | 0.016 |
| **High-recruiter status** | 6.952 | 2.302-20.945 | <0.001 |

BMI: body mass index; Crs: respiratory system compliance; FiO_2_: inspired fraction in oxygen; PaO_2_: partial arterial pressure in oxygen.

# Table S6. Respiratory and hemodynamic variables at baseline, end of prone position and post prone position.

|  | Pre-PP  (N=201) | PP  (N=201) | Post-PP  (N=137) |
| --- | --- | --- | --- |
| Respiratory variables | | | |
| Vt, mL/kg | 6.1±0.3 | 6.1±0.3 | 6.1±0.3 |
| RR, /min | 29±4 | 30±4 | 30±4 |
| Vm, L/min | 11.7±2.1 | 11.8±2.1 | 12.0±2.1 |
| PEEP, cmH_2_0 | 13±3 | 13±3 | 13±3 |
| PEEPt, cmH_2_0 | 14±3 | 14±3 | 14±3 |
| Pplat, cmH_2_0 | 29±4 | 28±4 | 29±4 |
| Driving pressure, cmH_2_0 | 15±5 | 14±5* | 15±6 |
| Crs, mL/cmH_2_0 | 30±11 | 33±13* | 31±9* |
| pH | 7.38±0.06 | 7.38±0.07 | 7.36±0.08 |
| pCO2, mmHg | 45±7 | 46±9 | 45±8 |
| PO_2_/FiO_2_, mmHg | 109±31 | 163±65* | 135±54* |
| AOP (N=156), mmHg | 0 (0-6) |  | 3 (0-6) |
| R/I (N=156) | 0.53 (0.30-0.76) | 0.51 (0.3-0.74) | 0.53 (0.27-0.83) |
| Hemodynamic variables | | | |
| HR, /min | 79±20 | 81±20 | 78±22 |
| SAP, mmHg | 134±49 | 130±21 | 135±24 |
| DAP, mmHg | 65±12 | 64±10 | 65±12 |
| MAP, mmHg | 88±16 | 87±13 | 89±16 |
| CVP (N=157), mmHg | 11±4 | 11±4 | 12±4 |
| PPV, % | 7 (5-12) | 7 (5-10) | 6 (4-11) |
| CI, L/min/m^2^ | 3.0±0.9 | 3.0±0.9 | 2.9±0.9 |
| GEDVI, mL/m^2^ | 795±200 | 795±201 | 786±194 |
| EVLWi, mL/kg | 19±6 | 19±5 | 19±6 |
| PVPi | 3.7±1.2 | 3.7±1.2 | 3.7±1.2 |
| CFI, /min | 4.2±1.3 | 4.1±1.2 | 4.2±1.3 |
| Norepinephrine (N) | 153/200 | 145/200 | 109/143 |
| Norepinephrine dosage, µg/kg/min | 0.17 (0.09-0.33) | 0.17 (0.08-0.32) | 0.17 (0.08-0.34) |
| Lactate, mmol/L | 1.7 (1.2-2.3) | 1.8 (1.3-2.5) | 1.8 (1.4-2.6) |

AOP: airway opening pressure; CFI: cardiac function index; CI: cardiac index; Crs: respiratory system compliance; CVP: central venous pressure; DAP: diastolic arterial pressure; EVLWi: extravascular lung water index; FiO_2_: inspired fraction in oxygen; GEDVi: global end-diastolic volume index; HR: heart rate; MAP: mean arterial pressure; pCO_2_: partial arterial pressure in carbon dioxide; PEEP: positive end-expiratory pressure: PEEPt: total positive end-expiratory pressure; pO_2_: partial arterial pressure in oxygen; Pplat: plateau pressure; PPV: pulse pressure variation; PVPI: pulmonary vascular resistance index; RR: respiratory rate; R/I: recruitment-to-inflation ratio; SAP: systolic arterial pressure; Vt: tidal volume.

“Pre-PP”: ≤1 hour before PP, “PP”: at the end of the PP session, “Post-PP”: 6 to 8 hours after return in supine position.

*p<0.05 vs. previous position

# Table S7. Changes in respiratory variables with prone position and after prone position

|  | Pre-PP | PP | Post-PP | p-value |
| --- | --- | --- | --- | --- |
| PaO_2_/FiO_2_ (n=137), mmHg  Low-recruiters (n=51)  High-recruiters (n=51) | 111 ± 3  115 ± 5  111 ± 4 | 168 ± 5*^¶^  164 ± 9*^¶^  174 ± 10*^¶^ | 135 ± 5^ǂ^  129 ± 7  139 ± 9^ǂ^ | <0.001  <0.001  <0.001 |
| Crs (n=137), mL/cmH_2_0  Low-recruiters (n=48)  High-recruiters (n=46) | 31 ± 1  31 ± 2  34 ± 2 | 34 ± 1*^¶^  31 ± 2  39 ± 2*^¶^ | 31 ± 1  30 ± 1  33 ± 1 | 0.001  0.927  0.002 |
| Driving pressure (n=137), cmH_2_O  Low-recruiters (n=51)  High-recruiters (n=49) | 15 ± 0  15 ± 1  13 ± 1 | 14 ± 0*  15 ± 1  12 ± 1*^¶^ | 15 ± 1  15 ± 1  14 ± 1 | 0.018  0.753  0.008 |
| R/I (n=76)  Low-recruiters (n=35)  High-recruiters (n=41) | 0.58 ± 0.05  0.29 ± 0.03  0.84 ± 0.07 | 0.55 ± 0.05  0.51 ± 0.07*  0.58 ±0.06* | 0.61 ± 0.05  0.55 ± 0.08^ǂ^  0.66 ± 0.06 | 0.336  0.006  0.017 |

Values are expressed as Mean ± Standard error of mean. p-value is given for the ANOVA model

Crs: respiratory system compliance; pO_2_: partial arterial pressure in oxygen; R/I: recruitment-to-inflation ratio.

*p<0.05 pre-PP vs. PP, ^¶^ p<0.05 PP vs. Post-PP, ^ǂ^ p<0.05 pre-PP vs. post-PP.

# Figure S1. Flowchart


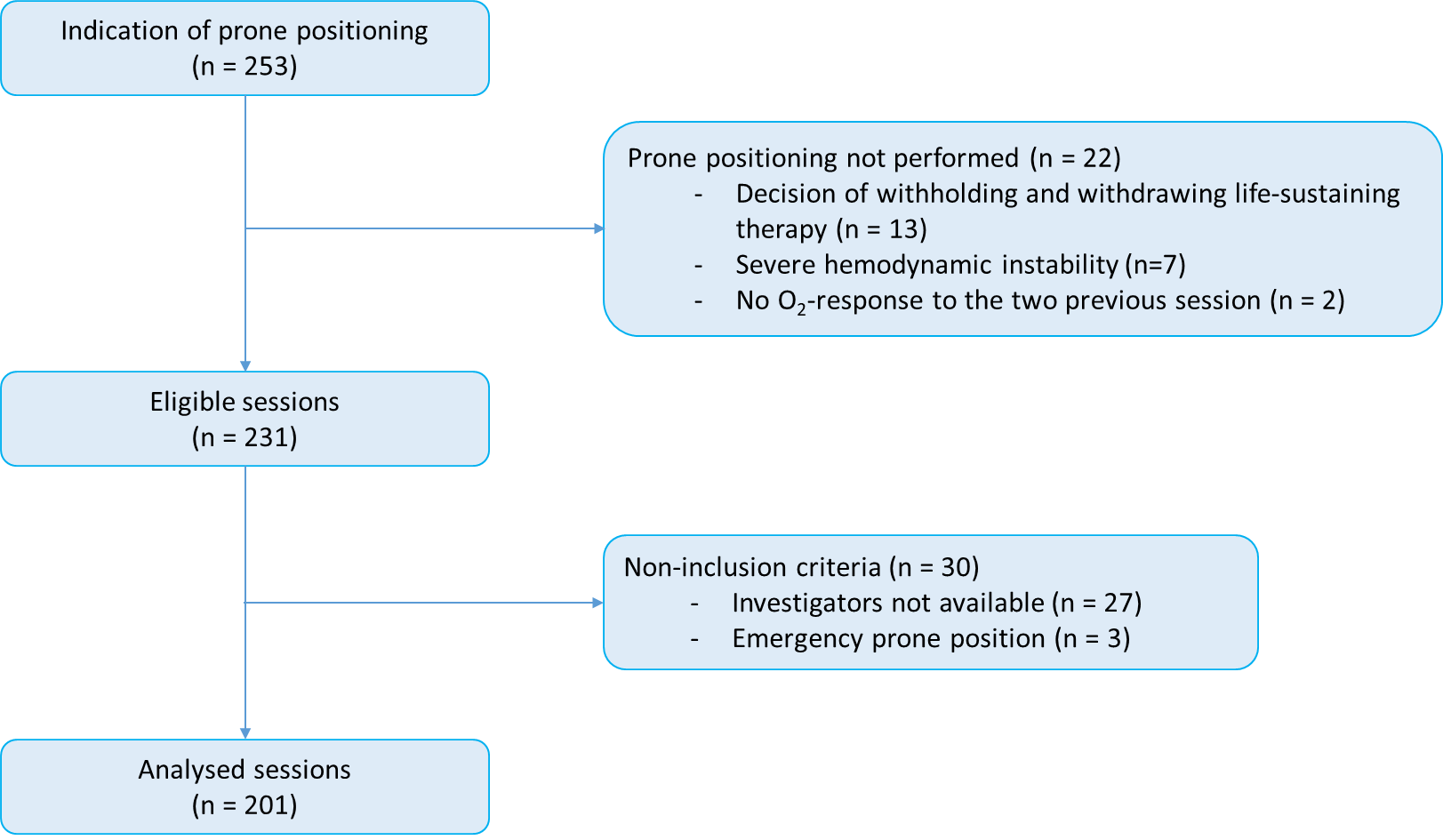


O_2_: *oxygen*

Figure S2. Alluvial diagram of patients’ movements between O2-response with prone position

(0: non- O_2_-responder; 1: O_2_-responder; MD: missing data) and patient outcome (red: deceased; blue: alive). Each solid bar represents an O_2_-response group at a given number of days since initiation of invasive mechanical ventilation. Shaded colored streams between bars represent transitions of patients between the O_2_-response groups from one-time point to the next, which is either their O_2_-response at the next session or their outcome. The height of the bars represents the proportion of patients at that time point (i.e., they stack up to 100%) and the height of a stream field represents the size of the components contained in both bars connected by the stream.

MD: missing data; O2-NR: oxygen-non-responder; O2-R: oxygen-responder


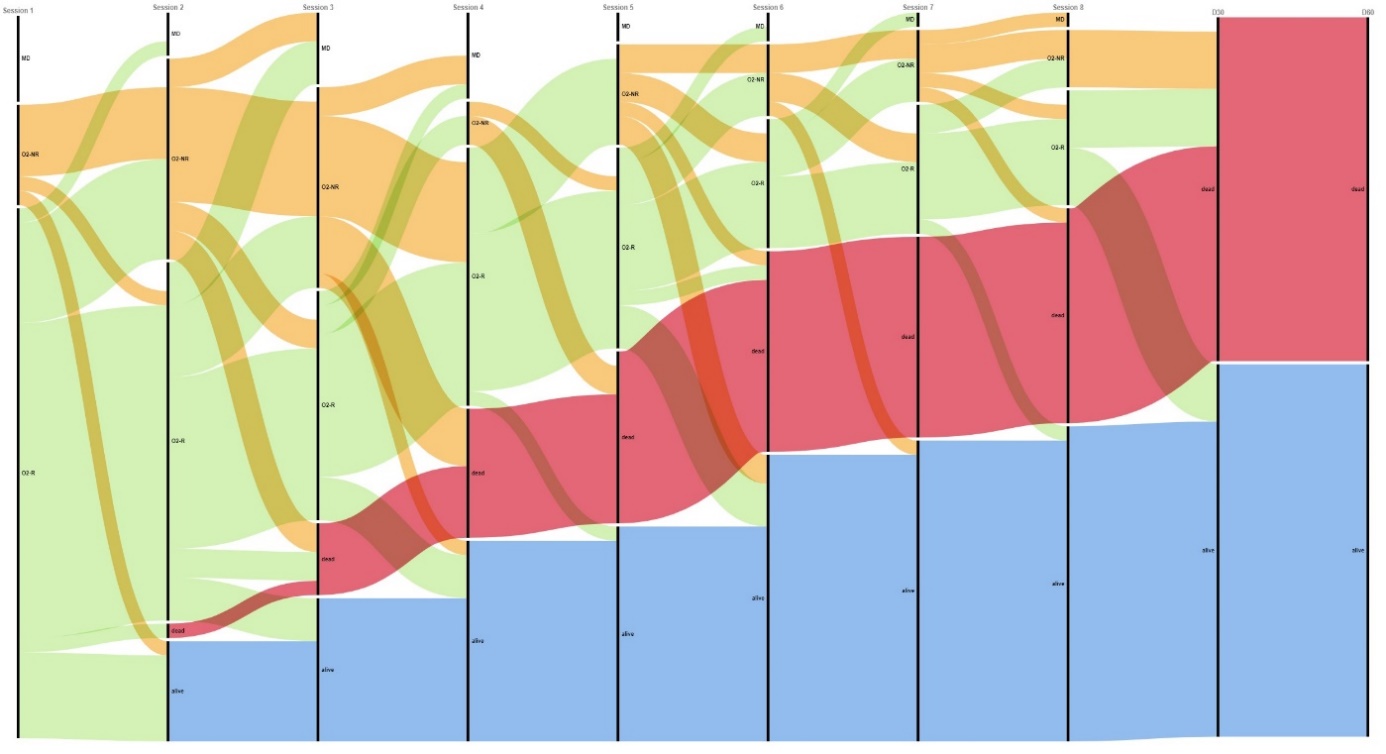


# Figure S3. Changes in lung mechanics and oxygenation with prone position and post prone position according to higher- and lower-recruiter profile.

Panel A: change in R/I; Panel B: change in PaO_2_/FiO_2_; Panel C: change in driving pressure; Panel D: change in respiratory system compliance.

*p<0.05: PP *vs*. pre-PP; ** p<0.05: PP *vs*. post-PP ; ^$^ p<0.05: pre-PP vs. post-PP

Crs: respiratory system compliance; DP: driving pressure; FiO_2_: fraction inspired in oxygen; PaO_2_: partial arterial pressure in oxygen; R/I: recruitment-to-inflation ratio.


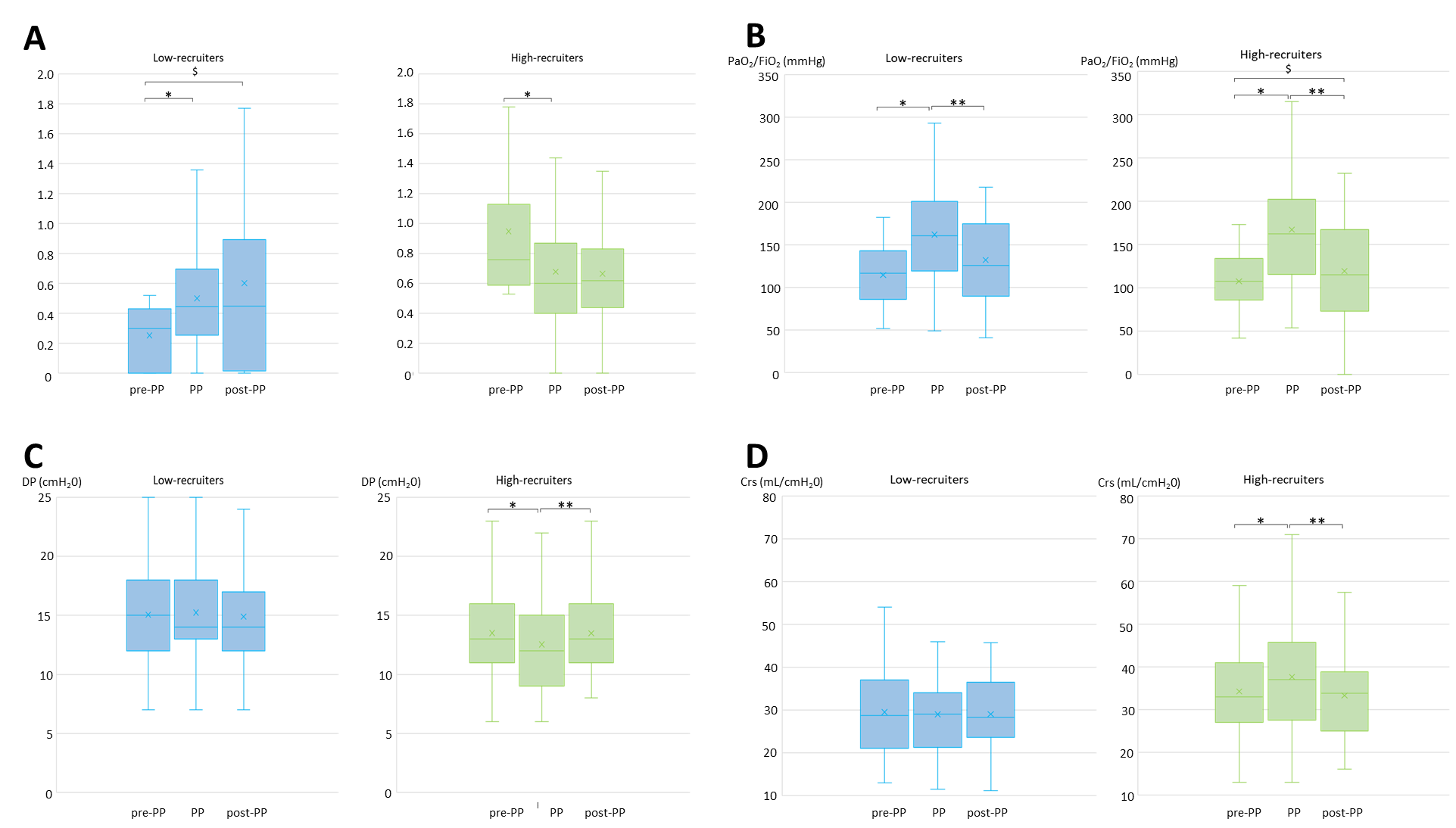


# Figure S4. Changes in airway opening pressure between two consecutives prone position sessions

0

2

4

6

8

10

12

14

Before previous PP session

Before next PP session

AOP (mmHg)

N = 86

AOP: airway opening pressure; PP: prone position.
